# Associations between Vegetable Nitrate Intake and Cardiovascular Disease Risk and Mortality: A Systematic Review

**DOI:** 10.3390/nu16101511

**Published:** 2024-05-17

**Authors:** Loucas Tan, Libby Stagg, Emily Hanlon, Toby Li, Andrea M. Fairley, Mario Siervo, Jamie Matu, Alex Griffiths, Oliver M. Shannon

**Affiliations:** 1 School of Biomedical, Nutritional & Sports Sciences, Newcastle University, Newcastle upon Tyne NE2 4HH, UK; c.l.tan2@newcastle.ac.uk (L.T.); l.a.stagg1@newcastle.ac.uk (L.S.); e.hanlon1@newcastle.ac.uk (E.H.); m.y.li2@newcastle.ac.uk (T.L.); andrea.fairley@newcastle.ac.uk (A.M.F.); 2 School of Population Health, Curtin University, Perth, WA 6845, Australia; mario.siervo@curtin.edu.au; 3 Curtin Dementia Centre of Excellence, Enable Institute, Curtin University, Perth, WA 6845, Australia; 4 School of Health, Leeds Beckett University, Leeds LS1 3HE, UK; j.matu@leedsbeckett.ac.uk (J.M.); a.griffiths@leedsbeckett.ac.uk (A.G.); 5 Human Nutrition & Exercise Research Centre, Centre for Healthier Lives, Population Health Sciences Institute, Newcastle University, Newcastle upon Tyne NE2 4HH, UK

**Keywords:** dietary nitrate, vegetables, cardiovascular disease, systematic review

## Abstract

Consumption of nitrate-rich vegetables increases nitric oxide bioavailability, lowers blood pressure, and improves endothelial function. These effects could also translate into reduced cardiovascular disease (CVD) risk and mortality. This systematic review aimed to investigate the associations between habitual vegetable nitrate intake and CVD incidence and mortality. A secondary aim was to identify factors that moderate the relationship between vegetable nitrate intake and CVD incidence/mortality. Seven databases (PubMed, MEDLINE, Embase, Scopus, Web of Science, CINAHL, and APA PsycINFO) were searched from inception to 13 February 2023. Observational studies quantifying vegetable nitrate intake in participants aged 18+ years through self-reported dietary exposure and assessing incidence or mortality from CVD overall, or individual CVD subtypes, were eligible. Five studies including a total of 63,155 participants were included. There was an inverse association between vegetable nitrate intake and most reported CVD outcomes. Reported risk reductions tended to plateau at moderate intake, suggesting a possible ceiling effect. The risk of bias across all studies was low. The results of this systematic review suggest a potential role for vegetable nitrate in reducing CVD risk and mortality. Further randomised controlled trials are now required to corroborate these findings.

## 1. Introduction

Cardiovascular disease (CVD) encompasses multiple disorders affecting the heart and blood vessels. It is the greatest contributor to global mortality, accounting for 32% of deaths annually, and the leading cause of global disease burden through the loss of disability-adjusted life years [1,2]. The major cause of CVD is atherosclerosis, resulting in stenosis of blood vessels and impairment of blood flow in the body [3]. The aetiology of atherosclerosis is multi-factorial, including risk factors such as elevated blood pressure (BP), endothelial dysfunction, and platelet aggregation [3,4]. Whilst elevated BP can be a significant contributor towards atherosclerosis and CVD risk, a 5 mmHg reduction in systolic BP has been associated with a 10% reduction in CVD risk, regardless of baseline BP [5].

The World Health Organisation estimates that ~80% of CVDs are preventable through modifying behavioural risk factors including diet [6]. Greater intake of vegetables (4–5 servings/day) is a cornerstone of cardioprotective diets and national interventions to combat CVD risk [7,8]. Analyses of large-scale cohort studies including the Nurses’ Health Study and Health Professionals Follow-up Study have highlighted a particularly strong inverse association between green leafy vegetable intake and CVD risk [9]. Specifically, each one serving increment of green leafy vegetables was associated with an 11% reduced risk of CVD when findings from both studies were combined [7,9]. The beneficial associations observed between vegetable intake and cardiovascular health may be related to the provision of inorganic nitrate, a dietary compound abundantly available in vegetables (especially green leafy vegetables) with promising cardioprotective effects [4,10].

Nitrate is a phytochemical abundant in the roots, leaves, and stems of vegetables, with vegetables contributing to 60–80% of daily nitrate intake [11]. Historically, at best, dietary nitrate was regarded as physiologically inert; at worst, it was thought to be harmful, potentially increasing risk of endocrine disorders, cancer, and infant methemoglobinemia [12,13]. The more-recent literature suggests that dietary nitrate intake could have cardioprotective effects, which appear to be related to the enhanced production of nitric oxide (NO) in the body [3,4,10]. Indeed, evidence from randomised controlled trials (RCTs) suggests that consumption of dietary nitrate in the form of vegetables, which elevates NO bioavailability, can influence multiple markers of cardiovascular health, lowering BP [4,14,15,16], decreasing platelet aggregation [15,17], and improving endothelial function [18,19]. Nevertheless, these studies typically include small sample sizes and are relatively short (days to weeks) in duration, such that they are not suitable for exploring the impact of dietary nitrate on CVD incidence or mortality.

The beneficial effects of vegetable-derived nitrate on markers of cardiovascular health could also translate into a reduction in CVD risk and mortality, with findings from some recent observational studies supporting this notion (e.g., [20,21,22]). Despite the recent growth in research in this area, there are currently no systematic reviews of this body of literature. However, a systematic review of investigations exploring the associations between vegetable nitrate intake and CVD incidence/mortality would be valuable to (1) outline the current state of knowledge in this area, (2) identify factors that might moderate associations between nitrate intake and CVD incidence/mortality, and (3) map out areas for future research. Against this background, we aimed to conduct the first systematic review to explore associations between habitual vegetable nitrate intake and CVD incidence and mortality.

## 2. Materials and Methods

The protocol for this systematic review was prospectively registered with PROSPERO (CRD42023393943). The review is reported following the Preferred Reporting Items for Systematic Reviews and Meta-Analyses (PRISMA) [23] and Synthesis Without Meta-Analysis (SWiM) guidelines [24].

### 2.1. Literature Search

Seven electronic databases (PubMed, MEDLINE, Embase, Scopus, Web of Science, CINAHL and APA PsycINFO) were comprehensively searched from database inception to 13 February, 2023 for relevant articles. Search terms related to vegetable nitrate intake and CVD incidence/mortality were utilised (Supplementary Material S1), with specific tailoring of terms for each database. MeSH terms were used where necessary. Reference lists of retrieved, eligible studies were also searched for potentially relevant articles. Only studies published in English were eligible for inclusion due to a lack of resource and expertise in other languages within the research team.

### 2.2. Study Selection

The following criteria were used to identify studies eligible for inclusion in this review:

**Participant:** Only studies including participants aged ≥18 years were eligible. Where studies included other age groups (e.g., children/adolescents), studies were only eligible for inclusion if the data on adults could be disaggregated.

**Exposure:** studies were eligible for inclusion if habitual vegetable nitrate intake was assessed using self-reported dietary assessment tools (e.g., food frequency questionnaires [FFQs], diet histories, and diet recalls).

**Comparison:** studies were included if they compared associations between higher and lower nitrate reference groups, as defined by the study authors (e.g., where the data were presented as tertiles, the lowest tertile of nitrate intake were considered the reference/comparator group against which other groups were be compared).

**Outcome:** studies were required to present data on overall CVD incidence or mortality (co-primary outcomes) or CVD subtype incidence or mortality (co-secondary outcomes).

**Study Design:** observational studies were eligible for inclusion. No further restrictions will be placed on the study design.

### 2.3. Selection Process

The titles and abstracts of the retrieved articles were screened in duplicate by two independent reviewers (from LT, TL, LS, and EH) to determine eligibility for inclusion. If there was agreement between both reviewers, articles were either moved to the next stage for full-text appraisal (if deemed to be potentially eligible for inclusion) or excluded from the review. If consensus was not reached, an additional independent reviewer (OMS) was consulted to decide on the best course of action. The full texts of the selected articles were then appraised in duplicate by two independent reviewers (from LT, TL, LS, and EH) to evaluate eligibility for inclusion in the systematic review. Again, if consensus was not reached, an additional independent reviewer (OMS) was consulted for dispute resolution. Screening was conducted using an online web tool, Rayyan [25]. Reasons for discarding articles at the full-text evaluation stage were recorded (Supplementary Table S4).

### 2.4. Data Extraction

Data from eligible articles were extracted utilising a standardised pre-piloted extraction form (Supplemental Table S1) created by the research team in Microsoft Excel. Data from each article were independently extracted in duplicate by two members of the research team (from LT, TL, LS, and EH), which were cross-compared to ensure accuracy. In cases of disagreement between the reviewers, an independent reviewer (OMS) was consulted. Data were extracted on study characteristics, demographics, exposure, and outcome data. The full list of data items is available in Supplemental Table S1.

### 2.5. Risk of Bias

The risk of bias in each eligible article was assessed by two independent reviewers (from LT, TL, LS, and EH) using the ROBINS-E tool [26]. This tool addresses seven domains of risk of bias: “(1) arising from confounding; (2) resulting from exposure measurement; (3) from selection of participants into the study/analysis; (4) arising from post-exposure interventions; (5) arising from missing data; (6) resulting from outcome measurement and (7) from selection of the reported result” [26]. As per previous stages of the review, in cases of disagreement between the reviewers, an independent reviewer (OMS) was consulted.

### 2.6. Evidence Synthesis

Given the small number of eligible studies and notable between-study heterogeneity, meta-analysis was deemed to be inappropriate for this study and a narrative synthesis of data was conducted [24]. Any effect measure that characterised risk estimates and CVD outcomes including hazard or odds ratios were synthesised and are presented in text and tables corresponding to vegetable nitrate intake reference groups (tertiles, quartiles, and quintiles) defined by study authors. Subgroup analyses of study-level variables were performed to assess if differences between studies (e.g., study design and participant characteristics) influenced reported associations.

## 3. Results

### 3.1. Overview

**Figure 1:**
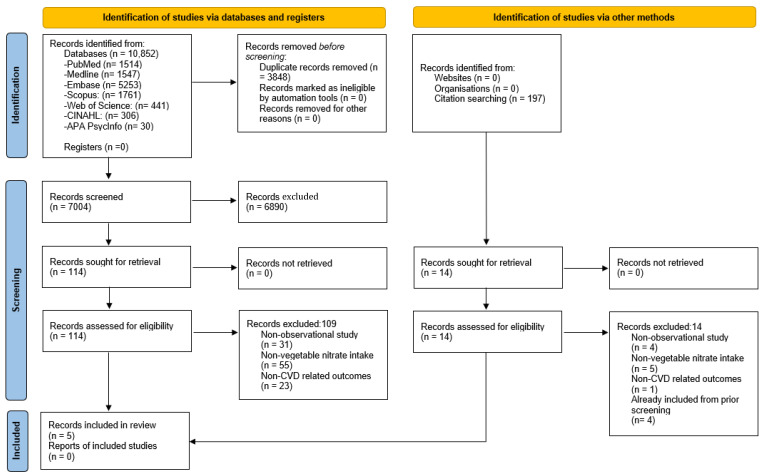
PRISMA flow diagram of studies included in this systematic review.

A total of 10,852 articles were identified across seven databases and reduced to 7004 after deduplication. A total of 6890 articles were excluded during title and abstract screening, resulting in 114 articles eligible for full-text screening. A total of 109 articles were subsequently excluded due to either having a non-observational study design or focusing on intake of non-vegetable nitrate or non-CVD outcomes. Five articles met the inclusion criteria. No additional articles were identified from the reference lists of retrieved articles (Figure 1).

### 3.2. Study Characteristics

The characteristics of the included studies are summarised in Table 1. All included studies had a prospective cohort design [21,22,27,28,29]. Four studies were conducted in Australia [22,27,28,29] with the remaining study in Denmark [29]. Two studies [22,29] included participants of both sexes, whilst the remaining three studies [21,27,28] only included female participants. All studies used validated semi-quantitative FFQs to quantify habitual vegetable nitrate intake. Studies by Bondonno et al. [21] and Bondonno et al. [29] quantified vegetable nitrate intake only once at baseline. All other studies [22,27,28] quantified vegetable nitrate intake at baseline and two future timepoints. Only Jackson et al. [27] assessed self-reported CVD outcomes. All other studies assessed CVD incidence/mortality via data linkage (e.g., to patient and hospital records/death registries).

### 3.3. Primary Outcomes

#### 3.3.1. Overall CVD Incidence

Two studies assessed overall CVD incidence. Bondonno et al. [29] reported that quintiles 2–5 of vegetable nitrate intake (39 mg/d; 59 mg/d; 92 mg/d; and 141 mg/d) were associated with 9%, 15%, 14%, and 14% risk reduction in overall CVD incidence, respectively. These associations were stronger in participants (quintile 5) with an average alcohol intake over 20 g/day (22% risk reduction) when compared to participants with an average intake below 20 g/day (8% risk reduction). Meanwhile, in the study by Jackson et al. [27], quartiles 2–4 (48.3 mg/d; 85 mg/d; and 117.3 mg/d) for vegetable nitrate intake were associated with 11%, 15%, and 27% risk reduction, respectively, for self-reported, CVD-related events (Table 2).

#### 3.3.2. Overall CVD Mortality

Only Liu et al. [22] assessed overall CVD mortality and reported that vegetable nitrate intake quartiles 2–4 (85 mg/d; 117.3 mg/d and 188.7 mg/d) were associated with 47%, 49%, and 37% risk reduction in overall CVD mortality versus quartile 1 (Table 3). These associations remained significant when stratified by gender and age.

### 3.4. Secondary Outcomes

#### 3.4.1. CVD Subtype Incidence

Associations between vegetable nitrate intake and CVD subtype incidence were evaluated by Bondonno et al. [29], who reported data on associations between vegetable nitrate intake and ischemic heart disease (IHD), heart failure, ischemic stroke, peripheral artery disease (PAD), and atrial fibrillation (AF). Quintiles 2–5 for vegetable nitrate intake (39 mg/d; 59 mg/d; 92 mg/d; and 141 mg/d) were associated with 7%, 12%, 14%, and 15% risk reduction in IHD incidence, respectively. For heart failure incidence, quintiles 2–5 (39 mg/d; 59 mg/d; 92 mg/d; and 141 mg/d) were associated with 9%, 15%, 14%, and 14% risk reduction, respectively. For ischaemic stroke incidence, quintiles 2–5 (39 mg/d; 59 mg/d; 92 mg/d; and 141 mg/d) were associated with 12%, 17%, 14%, and 13% risk reduction, respectively. For PAD, quintiles 2–5 (39 mg/d; 59 mg/d; 92 mg/d; and 141 mg/d) were associated with 16%, 26%, 30%, and 35% risk reduction, respectively. Finally, for AF incidence, quintiles 2–4 of vegetable nitrate intake (39 mg/d; 59 mg/d; and 92 mg/d) were associated with 6%, 9%, and 7% risk reduction, respectively (Table 4).

#### 3.4.2. CVD Subtype Mortality

Two studies [22,28] assessed CVD subtype mortality (Table 5). Blekkenhorst et al. [28] reported that tertiles 2–3 of vegetable nitrate intake (64.1 mg/d and 99.7 mg/d) were associated with 42% and 35% risk reduction for atherosclerotic vascular disease (ASVD) mortality, respectively.

Liu et al. [22] reported that quartiles 2–4 of vegetable nitrate intake (85.0 mg/d; 117.3 mg/d; and 188.7 mg/d) were associated with 54%, 27%, and 32% risk reduction for coronary heart disease (CHD) mortality, respectively. For stroke mortality, quartiles 2–4 (85 mg/d; 117.3 mg/d; and 188.7 mg/d) were associated with 34%, 82%, and 44% risk reduction, respectively.

#### 3.4.3. Combined Ischaemic Cerebrovascular Disease Hospitalisations and Mortality

Bondonno et al. [21] reported that tertiles 2–3 of vegetable nitrate intake (64.0 mg/d and 100 mg/d) were associated with a 29% and 35% risk reduction for ischaemic cerebrovascular disease hospitalisations or deaths, respectively.

All associations were derived comparing higher vegetable nitrate intake (quartile 2–4, quintile 2–5) with its referent group (quartile 4, quintile 5).

### 3.5. Risk of Bias Assessment

The risk of bias assessment of included studies is summarised in Supplemental Table S2. Domain-specific bias and overall risk of bias of all studies were deemed to be low. All studies controlled for pre-determined confounding factors in respective statistical models. All studies declared the possibility of residual confounding and unmeasured confounders as limitations.

## 4. Discussion

This study represents the first systematic review to explore associations between habitual vegetable nitrate intake and CVD incidence/mortality. The results of the five included studies suggest an inverse association between habitual vegetable nitrate intake and both incidence and mortality of overall CVD, alongside a reduction in incidence and mortality for most reported CVD subtypes. Collectively, the results suggest that higher vegetable nitrate intake is associated with improved cardiovascular health, which broadly agrees with the findings from a systematic review of RCTs on CVD risk factors [4], where vegetable nitrate supplementation reduced BP and arterial stiffness and improved endothelial function.

The greatest risk reductions for overall CVD incidence (15%) [29] and mortality (49%) [22] were associated with moderate vegetable nitrate intakes (quintile 3 and quartile 3, respectively) with no further benefits associated with higher intakes. These beneficial associations were achieved through higher mean habitual vegetable nitrate intakes of 36 mg/d and 69 mg/d for overall CVD incidence and mortality when compared to quintile and quartile 1. Such intakes could be achieved via a modest increase in consumption of nitrate-rich vegetables (e.g., 34.8 g and 66.6 g of lettuce or 34.1 g and 65.3 g of spinach, on average) [30]. Similarly, the greatest risk reductions for most reported CVD subtypes were associated with moderate vegetable nitrate intake (tertile 2, quartile 2–3, quintile 3). These benefits were achieved through higher mean habitual vegetable nitrate intakes of 26.8 mg/d and 69 mg/d when compared to respective reference groups (equivalent to 25.9 g and 66.6 g of lettuce or 25.4 g and 65.3 g of spinach [30]). Following the recommended vegetable portion size of 80 g by the Food and Agriculture Organisation and WHO [31], these findings suggest that even small increases in habitual vegetable nitrate intake (<1 portion/day) can be beneficial in reducing overall CVD incidence and mortality. This is particularly important from a public health standpoint, as it shows that small, potentially realistic, and low-cost changes in diet [32,33] could be beneficial for CV health. Key vegetable sources of nitrate in the studies included several green leafy vegetables (e.g., lettuce, spinach, chard, and cabbage), alongside celery, beetroot, potatoes, pumpkin, green beans, broccoli, and carrots [21,22,27,28,29]. This demonstrates that a wide range of different vegetables could be consumed to boost nitrate intake, based on personal dietary preferences and availability.

A recent systematic review of RCTs [4] suggested the need for ~350–400 mg/d of vegetable nitrate to elicit cardiovascular benefits via short-term nitrate supplementation. However, the findings reported here suggests that such high doses might not be needed in the habitual diet to have longer-term benefits on cardiovascular health. This suggests there might be an interplay between the duration of nitrate intake and the dose required to elicit effects. Additional longer-term RCTs are warranted to confirm this notion. However, interestingly, one recent study suggested that moderate doses of nitrate over a 13-week intervention may have more pronounced effects on BP than higher doses [34] (contrasting the more linear dose–response highlighted in acute studies, e.g., [35]).

The beneficial associations reported here may be explained by the augmentation of NO bioavailability by dietary nitrate via the enterosalivary nitrate—nitrite—NO pathway and downstream effects of NO on various cardiovascular risk factors (e.g., BP, endothelial function, and platelet aggression) [4]. Findings from a systematic review of RCTs identified an overall reduction by 4.8 mmHg and 1.7 mmHg of resting systolic and diastolic BP, respectively, with inorganic nitrate intake [4]. These reductions are close to the levels estimated to reduce mortality from overall CVD, stroke, and CHD by 7%, 14%, and 9%, respectively [4]. The respective risk reductions identified in this review are considerable (CVD mortality: 37–49%, stroke mortality: 34–82%, and CHD mortality: 27–54%) and may be due to vegetable nitrate impacting multiple CVD risk factors on top of BP.

This review has numerous strengths, including (1) pre-registration of the protocol on PROSPERO, thereby minimising reporting bias; (2) the close adherence to PRISMA and SWiM guidelines; and (3) our comprehensive database searches, which were designed in consultation with an information specialist, maximising the inclusivity of this review. This review also has certain limitations, including (1) only including studies published in the English language, which could result in the exclusion of some eligible articles; (2) not conducting a meta-analysis due to the limited number of studies identified and the variability in study outcomes reported—as more research on this topic emerges, meta-analysis of results should be undertaken to generate a pooled point estimate of effect sizes and to better understand dose–response (e.g., via meta-regression); and (3) although we set out to identify effect moderators, there were limited data on this across studies and only one potential effect moderator (level of alcohol intake) was identified [29].

There are also certain strengths and limitations to the primary literature identified. Strengths include (1) the use of validated dietary assessment tools to quantify vegetable nitrate intake; (2) controlling for multiple potential confounders in statistical analyses; and (3) the long duration of follow-up periods (minimum 14.5 years), providing long-term data on associations between vegetable nitrate intake and CVD incidence. Limitations include (1) the observational nature of the studies, which precludes cause–effect inferences from being made; (2) the risk of residual/unmeasured confounding; (3) the predominance of female participants across studies (although associations appeared to be similar in mixed-sex cohorts) and the lack of geographical variation (most studies were conducted in Australia), which limits generalisability; and (4) the inconsistency between studies for definitions of low, medium, and high-nitrate-intake groups, which makes identification of an optimal nitrate dose challenging.

## 5. Conclusions

The results of this systematic review suggest that vegetable nitrate intake is inversely associated with the incidence and mortality of most reported CVD outcomes. Benefits were apparent with modest increases in vegetable nitrate intake, suggesting that the addition of one portion per day of nitrate-rich vegetables (e.g., 80 g lettuce or spinach) to habitual diet could be an effective way to lower CVD risk and mortality. Longer-term, large-scale RCTs exploring the feasibility, acceptability, and efficacy of increasing vegetable nitrate intake are now warranted to substantiate these findings.

## Figures and Tables

**Table 1:** Summary of baseline characteristics of included observational studies.

Study	Country	Study Type	Study Period (y)	Sample Size (*n*)	Age (y) ^1^	BMI (kg/m^2^) ^1^	BP (mmHg)	Exposure Assessment and Frequency	Outcome Measure	Outcome Ascertainment
Bondonno et al. (2021) [29]	Denmark	Prospective cohort study	23	53,150 (28,468 females, 24,682 males) ^2^	56 [52, 60]	26 [23, 28]	Systolic: 138 [124, 152] Diastolic: 83 [76, 90]	Validated Danish FFQ at baseline	Overall CVD and CVD Subtype Incidence	ICD-10 codes using the DNPR
Liu et al. (2019) [22]	Australia	Prospective cohort study	15	2229 (1314 females, 915 males) ^2^	64.5 ± 9.1	26.0 ± 4.4	Systolic: 145.4 ± 20.8 Diastolic: 83.4 ± 9.8	Validated Willet FFQ at baseline, 5 y and 10 y timepoints	Overall CVD and CVD Subtype Mortality	Diagnosis codes (ICD-9 and ICD-10) using the ISCD
Blekkenhorst et al. (2017) [28]	Australia	Prospective cohort study	15	1226 (all female)	75.1 ± 2.7	27.0 ± 4.6	-	Validated Melbourne FFQ at 5 y and 7 y timepoints	CVD Subtype Mortality	Coded death certificates
Jackson et al. (2019b) [27]	Australia	Prospective cohort study	15	5324 (all female)	52.4 ± 1.4	25.0 ± 5.6	-	Validated DQES in 2001 and 2013	Overall CVD Incidence	Self-reported doctor diagnoses
Bondonno et al. (2017) [21]	Australia	Population-based study	≥14.5	1226 (all female)	75 ± 3	27 ± 5	-	Validated ACCV FFQ at baseline	Combined CVD Subtype Hospitalisation and Mortality	Retrieved from the Western Australian Data Linkage System

^1^ Data expressed as either mean ± SD or median [interquartile range]; ^2^ Data were unreported by study authors and calculated manually by current author; SD, Standard deviation; y, years; *n*, Numbers; BMI, Body mass index; BP, Blood pressure; FFQ, Food frequency questionnaire; ACCV, Anti-Cancer Council of Victoria; ICD, International Classification of Diseases; DQES, Dietary Questionnaire for Epidemiological Studies Version 2; ICD, International Classification of Diseases.

**Table 2:** Associations between vegetable nitrate intakes and CVD incidence.

Study	Total Participants Analysed	Total Vegetable Nitrate Intake (mg/d) ^1^	Outcome Occurrence *n* (%)	HR (95% CI) ^2,3^
Bondonno et al. (2021) [29]				
Overall CVD incidence ^4^				
Quintile 1 (Referent)	10,630	23 [18, 28]	3309 (31.1) ^5^	1.00 (Referent)
Quintile 2	10,630	39 [35, 43]	3037 (28.6) ^5^	0.91 (0.88, 0.94)
Quintile 3	10,630	59 [52, 68]	2725 (25.6) ^5^	0.85 (0.82, 0.89)
Quintile 4	10,630	92 [86, 98]	2456 (23.1) ^5^	0.86 (0.82, 0.89)
Quintile 5	10,630	141 [118, 168]	2561 (24.1) ^5^	0.86 (0.82, 0.90)
Total participants	53,150	59 [35, 98]	14,088 (26.5) ^5^	-
Jackson et al. (2019b) [27]				
Overall CVD-related events ^7^				
Quartile 1 (Referent)	1331	26.4 ± 11.3	505 (37.9) ^5^	1.00 ^6^ (Referent)
Quartile 2	1331	41.9 ± 7.4	504 (37.9) ^5^	0.89 ^6^ (0.78–1.02) (NS)
Quartile 3	1331	55.1 ± 9.1	495 (37.2) ^5^	0.85 ^6^ (0.73–0.98)
Quartile 4	1331	79.3 ± 22.3	447 (33.6) ^5^	0.73 ^6^ (0.61–0.88)
Total participants	5324	-	1951 (36.6)	-

^1^ Data expressed as median [Interquartile range], ^2^ HR (95% CI), unless stated otherwise, ^3^ All effect measures are statistically significant unless specified with ‘NS’ (not significant), ^4^ Incidence: hospitalisation with primary or secondary diagnosis, ^5^ Data on percentages were unreported by study authors and calculated to three significant figures manually by current authors, ^6^ Odds ratios (95% CI), ^7^ Self-reported outcomes. HR, hazard ratios; CI, confidence intervals; CVD, cardiovascular disease. All associations were derived comparing higher vegetable nitrate intake (quintile 2–5) with its referent group (quintile 1).

**Table 3:** Associations between vegetable nitrate intakes and overall CVD mortality.

Study	Total Participants Analysed	Total Vegetable Nitrate Intake (mg/d) ^1^	Outcome Occurrence *n* (%)	Effect Measure HR (95% CI) ^2,3^
Liu et al. (2019) [22]				
Quartile 1 (Referent)	557	48.3 ± 15.2	61 (11)	1.00 (Referent)
Quartile 2	558	85.0 ± 8.8	39 (7)	0.53 (0.35, 0.82)
Quartile 3	557	117.3 ± 10.9	35 (6.3)	0.51 (0.32, 0.80)
Quartile 4	557	188.7 ± 53.8	53 (9.5)	0.63 (0.41, 0.95)
Total participants	2229	109.7 ± 59.2	188 (8.4)	-

^1^ Data expressed as mean ± SD, ^2^ HR (95% CI), unless stated otherwise, ^3^ All effect measures are statistically significant, SD, standard deviation; HR, hazard ratios; CI, confidence intervals; CVD, cardiovascular disease. All associations were derived comparing higher vegetable nitrate intake (quartile 2–4) with its referent group (quartile 1).

**Table 4:** Associations between vegetable nitrate intakes and CVD subtype incidence.

Study	Total Participants Analysed	Total Vegetable Nitrate Intake (mg/d) ^1^	Outcome Occurrence *n* (%)	Effect Measure HR (95% CI) ^2,3^
Bondonno et al. (2021) [29]				
IHD incidence ^4^				
Quintile 1 (Referent)	10,630	23 [18, 28]	1279 (12.0) ^5^	1.00 (Referent)
Quintile 2	10,630	39 [35, 43]	1150 (10.8) ^5^	0.93 (0.88, 0.97)
Quintile 3	10,630	59 [52, 68]	1045 (9.83) ^5^	0.88 (0.82, 0.94)
Quintile 4	10,630	92 [86, 98]	846 (7.96) ^5^	0.86 (0.80, 0.93)
Quintile 5	10,630	141 [118, 168]	917 (8.63) ^5^	0.85 (0.79, 0.92)
Total participants	53,150	59 [35, 98]	5237 (9.85) ^5^	-
Bondonno et al. (2021) [29]				
Ischaemic stroke incidence ^4^				
Quintile 1 (Referent)	10,630	23 [18, 28]	714 (6.72) ^5^	1.00 (Referent)
Quintile 2	10,630	39 [35, 43]	613 (5.77) ^5^	0.88 (0.83, 0.94)
Quintile 3	10,630	59 [52, 68]	518 (4.87) ^5^	0.83 (0.76, 0.91)
Quintile 4	10,630	92 [86, 98]	509 (4.79) ^5^	0.86 (0.78, 0.94)
Quintile 5	10,630	141 [118, 168]	531 (5.00) ^5^	0.87 (0.78, 0.96)
Total participants	53,150	59 [35, 98]	2885 (5.43) ^5^	-
Bondonno et al. (2021) [29]				
Haemorrhagic stroke incidence ^4^				
Quintile 1 (Referent)	10,630	23 [18, 28]	170 (1.60) ^5^	1.00 (Referent)
Quintile 2	10,630	39 [35, 43]	127 (1.19) ^5^	0.91 (0.80, 1.04) (NS)
Quintile 3	10,630	59 [52, 68]	151 (1.42) ^5^	0.86 (0.71, 1.04) (NS)
Quintile 4	10,630	92 [86, 98]	121 (1.14) ^5^	0.87 (0.72, 1.06) (NS)
Quintile 5	10,630	141 [118, 168]	140 (1.32) ^5^	0.93 (0.76, 1.14) (NS)
Total participants	53,150	59 [35, 98]	709 (1.33) ^5^	-
Bondonno et al. (2021) [29]				
Heart failure incidence ^4^				
Quintile 1 (Referent)	10,630	23 [18, 28]	778 (7.32) ^5^	1.00 (Referent)
Quintile 2	10,630	39 [35, 43]	704 (6.62) ^5^	0.91 (0.85, 0.96)
Quintile 3	10,630	59 [52, 68]	575 (5.41) ^5^	0.85 (0.78, 0.93)
Quintile 4	10,630	92 [86, 98]	497 (4.68) ^5^	0.86 (0.78, 0.94)
Quintile 5	10,630	141 [118, 168]	527 (4.96) ^5^	0.86 (0.78, 0.95)
Total participants	53,150	59 [35, 98]	3081 (5.80) ^5^	-
Bondonno et al. (2021) [29]				
PAD incidence ^4^				
Quintile 1 (Referent)	10,630	23 [18, 28]	539 (5.07) ^5^	1.00 (Referent)
Quintile 2	10,630	39 [35, 43]	417 (3.92) ^5^	0.84 (0.78, 0.91)
Quintile 3	10,630	59 [52, 68]	363 (3.41) ^5^	0.74 (0.67, 0.83)
Quintile 4	10,630	92 [86, 98]	268 (2.52) ^5^	0.70 (0.62, 0.79)
Quintile 5	10,630	141 [118, 168]	255 (2.40) ^5^	0.65 (0.57, 0.74)
Total participants	53,150	59 [35, 98]	-	-
Bondonno et al. (2021) [29]				
AF incidence ^4^				
Quintile 1 (Referent)	10,630	23 [18, 28]	1457 (13.7) ^5^	1.00 (Referent)
Quintile 2	10,630	39 [35, 43]	1422 (13.4) ^5^	0.94 (0.90, 0.98)
Quintile 3	10,630	59 [52, 68]	1315 (12.4) ^5^	0.91 (0.85, 0.97)
Quintile 4	10,630	92 [86, 98]	1237 (11.6) ^5^	0.93 (0.87, 0.99)
Quintile 5	10,630	141 [118, 168]	1317 (12.4) ^5^	0.95 (0.89, 1.01) (NS)
Total participants	53,150	59 [35, 98]	6748 (12.7) ^5^	-

^1^ Data expressed as either mean ± SD or median [Interquartile range], ^2^ HR (95% CI), unless stated otherwise, ^3^ All effect measures are statistically significant, unless specified with (NS), ^4^ Incidence: hospitalisation with primary or secondary diagnosis, ^5^ Data on percentages were unreported by study authors and calculated to three significant figures manually by current authors. SD, standard deviation; HR, hazard ratios; CI, confidence intervals; CVD, cardiovascular disease; IHD, ischaemic heart disease; PAD, peripheral artery disease; AF, atrial fibrillation; NS, not statistically significant.

**Table 5:** Associations between vegetable nitrate intakes and CVD subtype mortality.

Study	Total Participants Analysed	Total Vegetable Nitrate Intake (mg/d) ^1^	Outcome Occurrence *n* (%)	Effect Measure HR (95% CI) ^2,3^
Blekkenhorst et al. (2017) [28]				
ASVD mortality				
Tertile 1 (Referent)	409	37.3 ± 11.0	102 (24.9)	1.00 (Referent)
Tertile 2	408	64.1 ± 6.7	61 (15)	0.58 (0.42, 0.82) *
Tertile 3	409	99.7 ± 20.8	75 (18.3)	0.65 (0.47, 0.92) *
Total participants	1226	67.0 ± 29.2	238 (19.4)	-
Overall trend per SD	1226	29.2 ± 0	-	0.79 (0.68, 0.93) ^#,^*
Liu et al. (2019) [22]				
CHD mortality				
Quartile 1 (Referent)	557	48.3 ± 15.2	37 (6.6)	1.00 (Referent)
Quartile 2	558	85.0 ± 8.8	22 (3.9)	0.46 (0.26, 0.82) ^#,1^
Quartile 3	557	117.3 ± 10.9	30 (5.4)	0.73 (0.43, 1.23) ^#,1^
Quartile 4	557	188.7 ± 53.8	36 (6.5)	0.68 (0.40, 1.15) ^#,1^
Total participants	2229	109.7 ± 59.2	125 (5.6)	-
Liu et al. (2019) [22]				
Stroke mortality				
Quartile 1 (Referent)	557	48.3 ± 15.2	24 (4.3)	1.00 (Referent)
Quartile 2	558	85.0 ± 8.8	17 (3)	0.66 (0.34, 1.26) ^#,2^
Quartile 3	557	117.3 ± 10.9	5 (0.9)	0.18 (0.06, 0.52) ^#,2^
Quartile 4	557	188.7 ± 53.8	17 (3.1)	0.56 (0.28, 1.11) ^#,2^
Total participants	2229	109.7 ± 59.2	63 (2.8)	-

^1^ Data expressed as mean ± SD, ^2^ HR (95% CI), unless stated otherwise, ^3^ All effect measures are statistically significant, * *p* trend 0.016, ^#^ *p* 0.004, ^#,1^ *p* 0.066, ^#,2^ *p* 0.014. SD, standard deviation; HR, hazard ratios; CI, confidence intervals; CVD, cardiovascular disease; ASVD, atherosclerotic vascular disease; CHD, coronary heart disease. All associations were derived comparing higher vegetable nitrate intake (tertile 2–3, quartile 2–4) with its referent group (tertile 1, quartile 1).

## Supplementary Materials

The following supporting information can be downloaded at: https://www.mdpi.com/article/10.3390/nu16101511/s1, Text S1: Search strategy; Table S1: Summary of risk of bias scores of included observational studies using the ROBINS-E tool; Table S2: Summary of methodological characteristics of included observational studies; Table S3: Articles excluded after full text screening with accompanying reasons (n = 109); Table S4: Articles excluded during citation screening of eligible articles with accompanying reasons (n = 14).
