# Supplementary material for: Associations between Vegetable Nitrate Intake and Cardiovascular Disease Risk and Mortality: A Systematic Review
Source: Nutrients. 2024 May 17;16(10):1511. doi: 10.3390/nu16101511 (PMC11124077; doi:10.3390/nu16101511)
Supplement: Supplementary file 1 [file nutrients-16-01511-s001.zip › nutrients-2997284-supplementary.pdf]

Supplementary material 1: Search strategy

Search strategies are presented as subject headings and syntax of respective databases for ease of generalisability for searches.

<u>MEDLINE (OVID) (1547 results)</u>

1. exp Cardiovascular Diseases/

2. cardiovascular disease*.mp.

3. CVD*.mp.

4. heart disease*.mp.

5. vascular disease*.mp.

6. cardiovascular infection*.mp.

7. exp Incidence/

8. incidence*.mp.

9. exp Mortality/

10. mortalit*.mp.

11. exp nitrates/ or exp nitrites/

12. nitrate*.mp. 13. 1 or 2 or 3 or 4 or 5 or 6 14. 7 or 8 or 9 or 10 15. 11 or 12 16. 13 and 14 and 15

<u>Embase (OVID) (5253 results)</u>

1. exp cardiovascular diseases/

2. "cardiovascular disease*".mp.

3. CVD*.mp.

4. exp incidence/

5. incidence*.mp.

6. exp mortality/

7. mortalit*.mp.

8. exp nitrate/

9. nitrate*.mp.

10. 1 or 2 or 3 11. 4 or 5 or 6 or 7 12. 8 or 9 13. 10 and 11 and 12

Scopus (1761 results)

(TITLE-ABS-KEY ("cardiovascular disease*" OR "CVD*" OR "heart disease*" OR "vascular disease*" OR "cardiovascular infection*") AND TITLE-ABS-KEY (incidence* OR mortalit*) AND TITLE-ABS-KEY (nitrate*))

Web of Science (441 results)

“cardiovascular disease*" OR CVD* OR "heart disease*" OR "vascular disease*" OR "cardiovascular infections*" (Topic) AND incidence* OR mortalit* (Topic) AND nitrate* (Topic)

CINAHL (EBSCO) (306 results)

1. (MH "Cardiovascular Diseases+")
2. "cardiovascular disease*" or cvd* or "heart disease*" OR "vascular disease*" OR "cardiovascular infections*"
3. (MH "Incidence")
4. "incidence*"
5. (MH "Prevalence")
6. "prevalence*"
7. (MH "Mortality+")
8. "mortalit*"
9. (MH "Death+")
10. "death*"
11. (MH "Nitrates")
12. “nitrate*"
13. ((S1 OR S2) AND (S3 OR S4 OR S5 OR S6 OR S7 OR S8 OR S9 OR S10)) AND (S11 OR S12)

APA PsychInfo (OVID) (30 results)

1. exp Cardiovascular Disorders/
2. "cardiovascular disease*".mp.
3. CVD*.mp.
4. "heart disease*".mp.

5. "vascular disease*".mp.

6. "cardiovascular infection*".mp.

7. exp Heart Disorders/

8. exp Epidemiology/

9. incidence*.mp.

10. 1exp "Death and Dying"/

11. 1exp Mortality Rate/

12. 1exp Mortality Risk/

13. 1mortality*.mp.

14. 1exp Nitric Oxide/

15. nitrate*.mp. 16. 1 or 2 or 3 or 4 or 5 or 6 or 7 17. 8 or 9 or 10 or 11 or 12 or 13 18. 14 or 15 19. 16 and 17 and 18

Supplementary Table 1. Summary of risk of bias scores of included observational studies using the ROBINS-E tool

Study	(1) Risk of bias due to confounding	(2) Risk of bias arising from measurement of the exposure	(3) Risk of bias in selection of participants into the study (or into the analysis)	(4) Risk of bias due to post-exposure interventions	(5) Risk of bias due to missing data	(6) Risk of bias from measurement of the outcome	(7) Risk of bias in selection of the reported result	Overall rating
**Overall CVD and CVD Subtype Incidence**								
Bondonno et al. (2021)	Low risk of bias, except for concerns about uncontrolled confounding	Low risk of bias	Low risk of bias	Low risk of bias	Low risk of bias	Low risk of bias	Low risk of bias	Low risk of bias
**Overall CVD and CVD Subtype Mortality**								
Liu et al. (2019)	Low risk of bias, except for concerns about uncontrolled confounding	Low risk of bias	Low risk of bias	Low risk of bias	Low risk of bias	Low risk of bias	Low risk of bias	Low risk of bias
**CVD Subtype Mortality**								
Blekkenhorst et al. (2017)	Low risk of bias, except for concerns about uncontrolled confounding	Low risk of bias	Low risk of bias	Low risk of bias	Low risk of bias	Low risk of bias	Low risk of bias	Low risk of bias
Jackson et al. (2019b)	Low risk of bias, except for concerns about uncontrolled confounding	Low risk of bias	Low risk of bias	Low risk of bias	Low risk of bias	Low risk of bias	Low risk of bias	Low risk of bias

Study	(1) Risk of bias due to confounding	(2) Risk of bias arising from measurement of the exposure	(3) Risk of bias in selection of participants into the study (or into the analysis)	(4) Risk of bias due to post-exposure interventions	(5) Risk of bias due to missing data	(6) Risk of bias from measurement of the outcome	(7) Risk of bias in selection of the reported result	Overall rating
**CVD Subtype Incidence and Mortality**								
Bondonno et al. (2017)	Low risk of bias, except for concerns about uncontrolled confounding	Low risk of bias	Low risk of bias	Low risk of bias	Low risk of bias	Low risk of bias	Low risk of bias	Low risk of bias

All included studies were assessed in accordance to the seven domains of the ROBINS-E tool (44).
Summarised bias scores for each included paper are available in Appendix 6.

Supplementary Table 2. Summary of methodological characteristics of included observational studies

Study	Statistical test	Main statistical model chosen	Covariates of main statistical model chosen[1]
**Overall CVD and CVD Subtype Incidence**			
Bondonno et al. (2021)	Cox proportional hazard models	Multivariable adjusted model (1b)	Age, sex, BMI, smoking status (current/former/never), physical activity (total daily metabolic equivalent), pure alcohol intake (g/day), social economic status (income), marital status, hypercholesterolemia (yes/no), education, and prevalent disease (diabetes, chronic obstructive pulmonary disease, chronic kidney disease and cancer
**Overall CVD and CVD Subtype Mortality**			
Liu et al. (2019)	Cox proportional hazard regression	Multivariable-adjusted model	Age (years), gender (female or male), BMI (kg/m2), physical activity (not active, light exercise or vigorous exercise), alcohol intake (grams per day), smoking history (never smoked, previous smoker or current smoker), socio-economic status (home/unit owner), use of organic nitrate medications (yes or no), use of antihypertensive medications (yes or no), use of statin medications (yes or no), use of low-dose aspirin (yes or no), renal function (MDRD eGFR, expressed as ml min^{-1} 1.73 m^{-2}) and energy intake (kcal per day).
**CVD Subtype Mortality**			
Blekkenhorst et al. (2017)	Cox proportional hazard models	Multivariable-adjusted model	Age, BMI, physical activity, alcohol intake, history of smoking, socioeconomic status, CAIFOS supplementation group, organic nitrate medication, antihypertensive medication, statin medication, low-dose aspirin, renal function and energy intake
Jackson et al. (2019b)	GEE models with time-varying covariates	Multivariate Model 3	Socio-economic status, education, vegetable intake (g/day) and energy (kJ/day)[2]
**CVD Subtype Incidence and Mortality**			
Bondonno et al. (2017)	Cox proportional hazard models	Baseline risk factor adjusted model	Age, BMI, energy intake, alcohol intake, energy expended in physical activity, antihypertensive medication, statin medication, low-dose aspirin medication, organic nitrate medication, history of smoking, and treatment

[1]Units of measurement were provided if stated by study authors
[2]Determined using a software program DAGitty (48)
BMI: Body mass index
MDRD eGFR: modification of diet in renal disease study equation, estimated glomerular filtration rate
Fully completed data extraction forms are available in Appendix 5.

Supplementary Table 3. Excluded Articles After Full Text Screening with Accompanying Reasons (n = 109)

Reason for Exclusion	Reference
Non-observational study design	Ahluwalia A, Hobbs AJ. Letter by Ahluwalia and Hobbs regarding article, "Nitrate-nitrite-nitric oxide pathway in pulmonary arterial hypertension therapeutics". *Circulation.* 2013;127(2): 275.
Non-observational study design	Abstracts from the 2018 Annual Scientific Meeting of the British and Irish Hypertension Society (BIHS). *Journal of Human Hypertension.* 2018;32(10): 693-721.
Non-observational study design	Ali H, Shahid M. Role of Compounds Present in Fruits and Vegetables in Preventing Heart Diseases. *Pakistan Heart Journal.* 2016;49(01): 03-8.
Non-observational study design	Allen KE, Billingsley HE, Carbone S. Nutrition, Heart Failure, and Quality of Life: Beyond Dietary Sodium. *Journal of the American College of Cardiology Heart Failure*. 2020;8(9): 765-769.
Non-observational study design	Ashworth A, Bescos R. Dietary nitrate and blood pressure: evolution of a new nutrient? *Nutrition Research Reviews.* 2017;30(2): 208-219.
Non-observational study design	Bahadoran Z, Mirmiran P, Azizi F, Ghasemi A. Nitrate-rich dietary supplementation during pregnancy: The pros and cons. *Pregnancy Hypertension.* 2018;10(5): 44-46.
Non-observational study design	Blekkenhorst LC, Sim M, Bondonno CP, Bondonno NP, Ward NC, Prince RL, Devine A, Lewis JR, Hodgson JM. Cardiovascular Health Benefits of Specific Vegetable Types: A Narrative Review. *Nutrients.* 2018;10(5): 595.
Non-observational study design	Bonilla Ocampo DA, Paipilla AF, Marín E, Vargas-Molina S, Petro JL, Pérez-Idárraga A. Dietary Nitrate from Beetroot Juice for Hypertension: A Systematic Review. *Biomolecules*. 2018;8(4): 134.[1]
Non-observational study design	Conor K. A16600 Nutritional Strategies to Prevent and Treat Heart Failure: A Comprehensive Review of Human Studies. *Journal of Hypertension.* 2018;36: 242-243.
Non-observational study design	Gilchrist M, Shore A. Inorganic Nitrate: Marker or Mediator of Mortality? *Journal of the American Heart Association.* 2017;6(11): e007782.
Non-observational study design	Hopper I, Connell C, Briffa T, De Pasquale CG, Driscoll A, Kistler PM, Macdonald PS, Sindone A, Thomas L, Atherton JJ. Nutraceuticals in Patients with Heart Failure: A Systematic Review. *Journal of Cardiac Failure.* 2020;26(2): 166-179.[1]
Non-observational study design	Ismaeel A, Greathouse KL, Newton N, Miserlis D, Papoutsi E, Smith RS, Eidson JL, Dawson DL, Milner CW, Widmer RJ, Bohannon WT, Koutakis P. Phytochemicals as Therapeutic Interventions in Peripheral Artery Disease. *Nutrients.* 2021;13(7): 2143.
Non-observational study design	Kerley CP. A Review of Plant-based Diets to Prevent and Treat Heart Failure. *Cardiac Failure Review.* 2018;4(1): 54-61.
Non-observational study design	Kerley CP. Dietary Patterns and Components to Prevent and Treat Heart Failure: A Comprehensive Review of Human Studies. *Nutrition Research Reviews.* 2019;32(1): 1-27.[1]
Non-observational study design	Mansor WA, Harith S, Taha CS. Effects of Various Dietary Pattern on Blood Pressure Management: A Review. *Malaysian Applied Biology.* 2019;48(4): 1-10.
Non-observational study design	McGrattan AM, Stephan BCM, Shannon OM, Mazidi M, Gilchrist M, Smallwood M, Winyard P, McMahon N, Blekkenhorst LC, Mohan D, Bandinelli S, Robinson L, Ferrucci L, Siervo M. Independent and Interactive Associations of Dietary Nitrate and Salt Intake with Blood Pressure and Cognitive Function: A Cross-Sectional Analysis in the InCHIANTI study. *International Journal of Food Sciences and Nutrition.* 2022;73(4): 491-502.
Non-observational study design	Mills C, Faconti L, Govoni V, Casagrande M-L, Maskell P, Masani A, Crickmore H, Iqbal F, Morant S, Webb A, Cruickshank K. Central Pressure and Cardiac Effects of Dietary Nitrate from Beetroot Juice in People with or at Risk of Type 2 Diabetes: The Randomised, Controlled VaSera Trial. *Journal of Human Hypertension.* 2017;31(10): 671.

Reason for Exclusion	Reference
Non-observational study design	Remington J, Winters K. Effectiveness of Dietary Inorganic Nitrate for Lowering Blood Pressure in Hypertensive Adults: A Systematic Review. *JBI Database of Systematic Reviews and Implementation Reports.* 2019;17(3): 365-389.
Non-observational study design	Saez O. Anabelle, Mitchell Anna, Philipp Thomas and Nurnberger Jens, Arterial Stiffness: A Potential Therapeutic Target to Reduce Cardiovascular Mortality. *Current Hypertension Reviews.* 2006; 2(2).
Non-observational study design	Shannon OM, Easton C, Shepherd AI, Siervo M, Bailey SJ, Clifford T. Dietary Nitrate and Population Health: A Narrative Review of the Translational Potential of Existing Laboratory Studies. *BMC Sports Science, Medicine and Rehabilitation.* 2021;13(1): 65.
Non-observational study design	Sleight P. Current options in the Management of Coronary Artery Disease. The American Journal of Cardiology. 2003;92(9B): 4-8.[1]
Non-observational study design	Spieker LE, Lüscher TF, Noll G. Current Strategies and Perspectives for Correcting Endothelial Dysfunction in Atherosclerosis. *Journal of Cardiovascular Pharmacology.* 2001;38(2): 35-41.[2]
Non-observational study design	Tucci M, Marino M, Martini D, Porrini M, Riso P, Del Bo' C. Plant-Based Foods and Vascular Function: A Systematic Review of Dietary Intervention Trials in Older Subjects and Hypothesized Mechanisms of Action. *Nutrients.* 2022;14(13): 2615.
Non-observational study design	Veerabhadrappa P, Schutte AE. Blood Pressure with Nitrate Exposure: Back-to-Basics with Fresh Fruits and Vegetables. *American Journal of Hypertension.* 2017;30(7): 665-666.
Non-observational study design	Wang Y, Chen W, Zhou J, Wang Y, Wang H, Wang Y. Nitrate Metabolism and Ischemic Cerebrovascular Disease: A Narrative Review. *Frontiers in Neurology.* 2022;13: 735181.
Non-vegetable nitrate intake	Al-Qallaf S M, Sayed Jalal Z. Tolerability and Compliance Among Patients using Long Acting Organic Nitrates. *International Journal of Pharmaceutical and Clinical Research.* 2017;9(5): 402-409.
Non-vegetable nitrate intake	Bahadoran Z, Mirmiran P, Jeddi S, Carlström M, Azizi F, Ghasemi A. Circulating Markers of Nitric Oxide Homeostasis and Cardiometabolic Diseases: Insights from Population-Based Studies. *Free Radical Research.* 2019;53(4): 359-376.
Non-vegetable nitrate intake	Bahadoran Z, Mirmiran P, Jeddi S, Momenan AA, Azizi F, Ghasemi A. The Nitrate-Nitrite-Nitric Oxide Pathway: Findings from 20 Years of the Tehran Lipid and Glucose Study. International Journal of *Endocrinology and Metabolism*. 2018;16(4): e84775.
Non-vegetable nitrate intake	Bahadoran Z, Mirmiran P, Tahmasebinejad Z, Azizi F, Ghasemi A. Serum nitric oxide metabolites and hard clinical endpoints: a population-based prospective study. *Scandinavian Cardiovascular Journal.* 2019;53(4): 176-182.
Non-vegetable nitrate intake	Björkegren JLM, Lusis AJ. Atherosclerosis: Recent Developments. *Cell.* 2022;185(10): 1630-1645.[1]
Non-vegetable nitrate intake	Blekkenhorst LC, Bondonno CP, Lewis JR, Devine A, Zhu K, Lim WH, Woodman RJ, Beilin LJ, Prince RL, Hodgson JM. Cruciferous and Allium Vegetable Intakes are Inversely Associated With 15-Year Atherosclerotic Vascular Disease Deaths in Older Adult Women. *Journal of the American Heart Association.* 2017;6(10): e006558.
Non-vegetable nitrate intake	Caro CG, Lever MJ. Water hardness, cardiovascular disease, and nitrate intake. Circulation. 1981;1(8210): 50.
Non-vegetable nitrate intake	Coggan AR, Peterson LR. Dietary Nitrate and Skeletal Muscle Contractile Function in Heart Failure. *Current Heart Failure Reports.* 2016;13(4): 158-65.
Non-vegetable nitrate intake	Cortez J. Atherosclerosis. Old problem, New Perspectives. *Acta Médica Portuguesa.* 2000;13(3): 101-5.[1]
Non-vegetable nitrate intake	Lim SL, Benson L, Dahlström U, Lam CS, Lund LH. Association Between Use of Long-Acting Nitrates and Outcomes in Heart Failure With Preserved Ejection Fraction. *Circulation: Heart Failure.* 2017;10(4): e003534.

Reason for Exclusion	Reference
Non-vegetable nitrate intake	Lisi M, Parker JD, Gori T. Organic Nitrates: Still Interesting After All These Years. *Recenti Progressi in Medicina.* 2009;100(3): 140-143.
Non-vegetable nitrate intake	Londono-Hoyos F, Zamani P, Beraun M, Vasim I, Segers P, Chirinos JA. Effect of Organic and Inorganic Nitrates on Cerebrovascular Pulsatile Power Transmission in Patients with Heart Failure and Preserved Ejection Fraction. *Physiological Measurement.* 2018;39(4): 044001.
Non-vegetable nitrate intake	Lovegrove JA, Stainer A, Hobbs DA. Role of flavonoids and nitrates in cardiovascular health. *Proceedings of the Nutrition Society.* 2017;19: 1-13.
Non-vegetable nitrate intake	Lundberg J, Gladwin M, Ahluwalia A, Benjamin N, Bryan NS, Butler A, *et al.* Nitrate and Nitrite in Biology, Nutrition and Therapeutics. *Nature Chemical Biology.* 2009;5: 865–869.
Non-vegetable nitrate intake	Maas R, Xanthakis V, Göen T, Müller J, Schwedhelm E, Böger RH, Vasan RS. Plasma Nitrate and Incidence of Cardiovascular Disease and All-Cause Mortality in the Community: The Framingham Offspring Study. *Journal of the American Heart Association.* 2017;6(11): e006224.[4]
Non-vegetable nitrate intake	Mendy A. Association of Urinary Nitrate with Lower Prevalence of Hypertension and Stroke and With Reduced Risk of Cardiovascular Mortality. *Circulation.* 2018;137(21): 2295-2297.[1]
Non-vegetable nitrate intake	Mirmiran P, Houshialsadat Z, Gaeini Z, Bahadoran Z, Azizi F. Functional Properties of Beetroot (Beta vulgaris) in Management of Cardio-metabolic Diseases. *Nutrition & Metabolism.* 2020;17: 3.
Non-vegetable nitrate intake	Mollenhauer M, Mehrkens D, Rudolph V. Nitrated fatty Acids in Cardiovascular Diseases. *Nitric Oxide.* 2018;1089-8603(17): 30292-30296.
Non-vegetable nitrate intake	Moschonis G, Karatzi K. Novel Dietary Approaches for Controlling High Blood Pressure. *Nutrients.* 2020;12(12): 3902.
Non-vegetable nitrate intake	Rammos C, Hendgen-Cotta UB, Sobierajski J, Bernard A, Kelm M, Rassaf T. Dietary Nitrate Reverses Vascular Dysfunction in Older Adults with Moderately Increased Cardiovascular Risk. *Journal of the American College of Cardiology.* 2014;63(15): 1584-1585.[1]
Non-vegetable nitrate intake	Ramms B, Gordts PL. Dietary Nitrate Struggles in Atherosclerosis. *Atherosclerosis.* 2016;245: 71-73.
Non-vegetable nitrate intake	Rathod KS, Jones DA, Van-Eijl TJ, Tsang H, Warren H, Hamshere SM, Kapil V, Jain AK, Deaner A, Poulter N, Caulfield MJ, Mathur A, Ahluwalia A. Randomised, Double-blind, Placebo-controlled Study Investigating the Effects of Inorganic Nitrate on Vascular Function, Platelet Reactivity and Restenosis in Stable Angina: Protocol of the NITRATE-OCT study. *BMJ Open.* 2016;6(12): e012728.[1]
Non-vegetable nitrate intake	Ryan-Woolley A, Cantrill J, Noyce P, Bennett E, Geoghegan M, Murphy M. A Partnership Between Community Pharmacists and General Practitioners in the Management of Ischaemic Heart Disease: A feasibility Study. *Pharmaceutical Journal.* 2001;267(7165): 355-358.
Non-vegetable nitrate intake	Saez O. Anabelle, Mitchell Anna, Philipp Thomas and Nurnberger Jens, Arterial Stiffness: A Potential Therapeutic Target to Reduce Cardiovascular Mortality. *Current Hypertension Reviews.* 2006; 2(2).
Non-vegetable nitrate intake	Shinohara Y, Yanagihara T, Abe K, Yoshimine T, Fujinaka T, Chuma T, Ochi F, Nagayama M, Ogawa A, Suzuki N, Katayama Y, Kimura A, Yasui N. III. Intracerebral Hemorrhage. *Journal of Stroke and Cerebrovascular Diseases.* 2011;20: S74-99.
Non-vegetable nitrate intake	Singh RB, Niaz MA, Ghosh S, Singh R, Rastogi SS. Effect on Mortality and Reinfarction of Adding Fruits and Vegetables to a Prudent Diet in the Indian experiment of Infarct Survival (IEIS). *Journal of the American College of Nutrition.* 1993;12(3): 255-61.
Non-vegetable nitrate intake	Slany J. Do Vasodilators Help in Chronic Heart Failure? *Wiener klinische Wochenschrift.* 1987;99(5): 139-144.
Non-vegetable nitrate intake	Sleight P. Current Options in the Management of Coronary Artery Disease. *American Journal of Cardiology. 2003*;92(9): 4-8.[1]
Non-vegetable nitrate intake	Smulyan H, Safar ME. The Diastolic Blood Pressure in Systolic Hypertension. *Annals of Internal Medicine.* 2000;132(3): 233-237.

Reason for Exclusion	Reference
Non-vegetable nitrate intake	Spieker LE, Lüscher TF, Noll G. Current Strategies and Perspectives for Correcting Endothelial Dysfunction in Atherosclerosis. *Journal of Cardiovascular Pharmacology.* 2001;38: S35-41.
Non-vegetable nitrate intake	Stock EO, Redberg R. Cardiovascular disease in women. *Current Problems in Cardiology.* 2012;(11): 450-526.
Non-vegetable nitrate intake	Storo, K.E., Harrison, C.D., Bockover, E.J., Ratcliffe, B.J., Crowe, S., Morales, F.J., Fair, H.M., Gurovich, A.N. & Carriker, C.R. Abstract P243: Endothelial Function, Arterial Stiffness and Central Blood Pressure Are Not Affected by a Single Dose of Dietary Nitrate in Healthy Normotensive Females. *Circulation.* 2018;137: 243.
Non-vegetable nitrate intake	Taggu W, Lloyd G. Treating Cardiovascular Disease in Women. *International Menopause Society.* 2007;13(4): 159-164.
Non-vegetable nitrate intake	Tenenbaum A, Fisman EZ, Boyko V, Goldbourt U, Graff E, Shemesh J, Shotan A, Reicher-Reiss H, Behar S, Motro M. Hypertension in Diet Versus Pharmacologically Treated Diabetics: Mortality over a 5-year Follow-up. *Hypertension.* 1999;33(4): 1002-1007.
Non-vegetable nitrate intake	Thadani U. Oral Nitrates: More than Symptomatic Therapy in Coronary Artery Disease? *Cardiovascular Drugs and Therapy.* 1997;11(1): 213-218.[1]
Non-vegetable nitrate intake	Tocci G, Ferrucci A, Guida P, Avogaro A, Comaschi M, Corsini A, Cortese C, Giorda CB, Manzato E, Medea G, Mureddu GF, Riccardi G, Titta G, Ventriglia G, Zito GB, Volpe M; EFFECTUS Steering Committee. An Analysis of the Management of Cardiovascular Risk Factors in Routine Clinical Practice in Italy: An Overview of the Main Findings of the EFFECTUS study. *High Blood Pressure & Cardiovascular Prevention.* 2011;18(1): 19-30.
Non-vegetable nitrate intake	Travessa AM, Menezes Falcão L. Vasodilators in Acute Heart Failure - Evidence Based on New Studies. *European Journal of Internal Medicine.* 2018;51: 1-10.
Non-vegetable nitrate intake	Veerabhadrappa P, Schutte AE. Blood Pressure With Nitrate Exposure: Back-to-Basics With Fresh Fruits and Vegetables. *American Journal of Hypertension.* 2017;30(7): 665-666.
Non-vegetable nitrate intake	Wong AP, Kassab YW, Mohamed AL, Abdul Qader AM. Review: Beyond Conventional Therapies: Complementary and Alternative Medicine in the Management of Hypertension: An Evidence-Based Review. *Pakistan Journal of Pharmaceutical Sciences.* 2018;31(1): 237-244.[2]
Non-CVD related outcomes	Ashfield-Watt PA. Fruits and vegetables, 5+ a Day: Are We Getting the Message Across? *Asia Pacific Journal of Clinical Nutrition.* 2006;15(2): 245-252.
Non-CVD related outcomes	Bahadoran Z, Mirmiran P, Ghasemi A, Carlström M, Azizi F, Hadaegh F. Association between Dietary Intakes of Nitrate and Nitrite and the Risk of Hypertension and Chronic Kidney Disease: Tehran Lipid and Glucose Study. *Nutrients.* 2016;8(12): 811.
Non-CVD related outcomes	Baliga RS, Milsom AB, Ghosh SM, Trinder SL, Macallister RJ, Ahluwalia A, Hobbs AJ. Dietary Nitrate Ameliorates Pulmonary Hypertension: Cytoprotective Role for Endothelial Nitric Oxide Synthase and Xanthine Oxidoreductase. *Circulation.* 2012;125(23): 2922-32.[1]
Non-CVD related outcomes	Crilly MA, McNeill G. Arterial Dysfunction in Patients with Rheumatoid Arthritis and the Consumption of Daily Fruits and Daily Vegetables. *European Journal of Clinical Nutrition.* 2012;66(3): 345-352.
Non-CVD related outcomes	Jackson JK, Patterson AJ, MacDonald-Wicks LK, Bondonno CP, Blekkenhorst LC, Ward NC, Hodgson JM, Byles JE, McEvoy MA. Dietary Nitrate and Diet Quality: An Examination of Changing Dietary Intakes within a Representative Sample of Australian Women. *Nutrients.* 2018;10(8): 1005.
Non-CVD related outcomes	Li X, Zhang W, Laden F, Curhan GC, Rimm EB, Guo X, Hart JE, Wu S. Dietary Nitrate Intake and Vegetable Consumption, Ambient Particulate matter, and Risk of Hypertension in the Nurses' Health study. *Environment International.* 2022;161: 107100.

Reason for Exclusion	Reference
Non-CVD related outcomes	Moreira LSG, Fanton S, Cardozo L, Borges NA, Combet E, Shiels PG, Stenvinkel P, Mafra D. Pink Pressure: Beetroot (Beta Vulgaris Rubra) as a Possible Novel Medical Therapy for Chronic Kidney Disease. *Nutrition Reviews.* 2022;80(5): 1041-1061.
Non-CVD related outcomes	Ntessalen M, Procter NEK, Schwarz K, Loudon BL, Minnion M, Fernandez BO, Vassiliou VS, Vauzour D, Madhani M, Constantin-Teodosiu D, Horowitz JD, Feelisch M, Dawson D, Crichton PG, Frenneaux MP. Inorganic Nitrate and Nitrite Supplementation Fails to Improve Skeletal Muscle Mitochondrial Efficiency in Mice and Humans. *The American Journal of Clinical Nutrition.* 2020;111(1): 79-89.
Non-CVD related outcomes	Omar SA, Artime E, Webb AJ. A Comparison of Organic and Inorganic Nitrates/Nitrites. *Nitric Oxide.* 2012;26(4): 229-40.[1]
Non-CVD related outcomes	Rammos C, Hendgen-Cotta UB, Sobierajski J, Bernard A, Kelm M, Rassaf T. Dietary Nitrate Reverses Vascular Dysfunction in Older Adults with Moderately Increased Cardiovascular Risk. *Journal of the American College of Cardiology.* 2014;63(15): 1584-1855.
Non-CVD related outcomes	Robinson, F. (2002) Five a Day - Still the Best Way. *Food and Nutrition Bulletin.* 2002;27(2): 83–84.
Non-CVD related outcomes	Shinohara Y, Yanagihara T, Abe K, Yoshimine T, Fujinaka T, Chuma T, Ochi F, Nagayama M, Ogawa A, Suzuki N, Katayama Y, Kimura A, Yasui N. III. Intracerebral Hemorrhage. *Journal of Stroke and Cerebrovascular Diseases.* 2011;20: S74-99.
Non-CVD related outcomes	Sim M, Lewis JR, Blekkenhorst LC, Bondonno CP, Devine A, Zhu K, Peeling P, Prince RL, Hodgson JM. Dietary Nitrate Intake is Associated with Muscle Function in Older Women. *Journal of Cachexia, Sarcopenia and Muscle.* 2019;10(3): 601-610.[1]
Non-CVD related outcomes	Singh RB, Niaz MA, Ghosh S, Singh R, Rastogi SS. Effect on Mortality and Reinfarction of Adding Fruits and Vegetables to a Prudent Diet in the Indian Experiment of Infarct Survival (IEIS). *Journal of the American College of Nutrition.* 1993;12(3): 255-61.
Non-CVD related outcomes	Smulyan H, Safar ME. The Diastolic Blood Pressure in Systolic Hypertension. *Annals of Internal Medicine.* 2000;132(3): 233-237.
Non-CVD related outcomes	Theodorakis NG, Wang YN, Korshunov VA, Maluccio MA, Skill NJ. Thalidomide Ameliorates Portal Hypertension via Nitric Oxide Synthase Independent Reduced Systolic Blood Pressure. *World Journal of Gastroenterology.* 2015; 21(14): 4126-4135.
Non-CVD related outcomes	Totzeck M, Hendgen-Cotta UB, Rassaf T. Nitrite-Nitric Oxide Signaling and Cardioprotection. *Advances in Experimental Medicine and Biology.* 2017;982: 335-346.
Non-CVD related outcomes	Veerabhadrappa, P. & Schutte, A.E. (2017) Blood Pressure with Nitrate Exposure: Back-to-basics with Fresh Fruits and Vegetables. *American Journal of Hypertension.* 2017;30(7): 665–666.
Non-CVD related outcomes	Volino-Souza M, de Oliveira GV, Conte-Junior CA, Alvares TS. Covid-19 Quarantine: Impact of Lifestyle Behaviours Changes on Endothelial Function and Possible Protective Effect of Beetroot Juice. *Frontiers in Nutrition.* 2020;7: 582210.[1]

[1]Two copies excluded
[2]Three copies excluded
[3]Four copies excluded
[4]Five copies excluded

Supplemental Table 4. Excluded Articles during Citation Screening of Eligible Articles With Accompanying Reasons (n = 14)

Reason for Exclusion	Reference
Non-observational study design	Bondonno CP, Blekkenhorst LC, Liu AH, Bondonno NP, Ward NC, Croft KD, Hodgson JM. Vegetable-derived Bioactive Nitrate and Cardiovascular Health. *Molecular Aspects of Medicine.* 2018;61: 83-91.
Non-observational study design	Hansen CP, Overvad K, Tetens I, Tjønneland A, Parner ET, Jakobsen MU, Dahm CC. Adherence to the Danish Food-based Dietary Guidelines and Risk of Myocardial Infarction: A Cohort Study. *Public Health Nutrition.* 2018;21(7): 1286-1296.
Non-observational study design	Jackson JK, Patterson AJ, MacDonald-Wicks LK, Oldmeadow C, McEvoy MA. The Role of Inorganic Nitrate and Nitrite in Cardiovascular Disease Risk Factors: A Systematic Review and Meta-Analysis of Human Evidence. *Nutrition Reviews.* 2018;76(5): 348-371.
Non-observational study design	Lidder S, Webb AJ. Vascular Effects of Dietary Nitrate (As Found in Green Leafy Vegetables and Beetroot) via the Nitrate-Nitrite-Nitric Oxide Pathway. *British Journal of Clinical Pharmacology.* 2013;75(3): 677-696.
Non-vegetable nitrate intake	Alissa EM, Ferns GA. Dietary Fruits and Vegetables and Cardiovascular Diseases Risk. *Critical Reviews in Food Science and Nutrition.* 2017;57(9): 1950-1962.
Non-vegetable nitrate intake	Bondonno CP, Croft KD, Hodgson JM. Dietary Nitrate, Nitric Oxide, and Cardiovascular Health. *Critical Reviews in Food Science and Nutrition.* 2016;56(12): 2036-52.
Non-vegetable nitrate intake	Jackson JK, Zong G, MacDonald-Wicks LK, Patterson AJ, Willett WC, Rimm EB, Manson JE, McEvoy MA. Dietary Nitrate Consumption and Risk of CHD in Women from the Nurses' Health Study. *British Journal of Nutrition.* 2019;121(7): 831-838.
Non-vegetable nitrate intake	Lundberg JO, Carlström M, Larsen FJ, Weitzberg E. Roles of dietary Inorganic Nitrate in Cardiovascular Health and Disease. *Cardiovascular Research.* 2011;89(3): 525-32.
Non-vegetable nitrate intake	Weitzberg E, Lundberg JO. Novel Aspects of Dietary Nitrate and Human Health. *Annual Review of Nutrition.* 2013;33: 129-159.
Non-CVD related outcomes	Jackson JK, Patterson AJ, MacDonald-Wicks LK, Bondonno CP, Blekkenhorst LC, Ward NC, Hodgson JM, Byles JE, McEvoy MA. Dietary Nitrate and Diet Quality: An Examination of Changing Dietary Intakes within a Representative Sample of Australian Women. *Nutrients.* 2018;10(8): 1005.
Already included from title and abstract screening	Bondonno CP, Blekkenhorst LC, Prince RL, Ivey KL, Lewis JR, Devine A, Woodman RJ, Lundberg JO, Croft KD, Thompson PL, Hodgson JM. Association of Vegetable Nitrate Intake with Carotid Atherosclerosis and Ischemic Cerebrovascular Disease in Older Women. *Stroke.* 2017;48(7): 1724-1729.
Already included from title and abstract screening	Blekkenhorst LC, Bondonno CP, Lewis JR, Devine A, Woodman RJ, Croft KD, Lim WH, Wong G, Beilin LJ, Prince RL, Hodgson JM. Association of Dietary Nitrate with Atherosclerotic Vascular Disease Mortality: A Prospective Cohort Study of Older Adult Women. *The American Journal of Clinical Nutrition.* 2017;106(1): 207-216.
Already included from title and abstract screening	Jackson JK, Patterson AJ, MacDonald-Wicks LK, Forder PM, Blekkenhorst LC, Bondonno CP, Hodgson JM, Ward NC, Holder C, Oldmeadow C, Byles JE, McEvoy MA. Vegetable Nitrate Intakes Are Associated with Reduced Self-Reported Cardiovascular-Related Complications within a Representative Sample of Middle-Aged Australian Women, Prospectively Followed up for 15 Years. *Nutrients.* 2019;11(2): 240.
Already included from title and abstract screening	Liu AH, Bondonno CP, Russell J, Flood VM, Lewis JR, Croft KD, Woodman RJ, Lim WH, Kifley A, Wong G, Mitchell P, Hodgson JM, Blekkenhorst LC. Relationship of Dietary Nitrate Intake from Vegetables with Cardiovascular Disease Mortality: A Prospective Study in a Cohort of Older Australians. *European Journal of Nutrition.* 2019;58(7): 2741-2753.
